# Department of Practice of Medicine

**Published:** 1895-10

**Authors:** 

**Affiliations:** Galveston


### Current Medical Literature.

### DEPARTMENT OF PRACTICE OF MEDICINE.

EDITED BY PROF. ALLEN J. SMITH, M. D., GALVESTON.

METHYLENE BLUE FOR CHYLURIA FROM FILARIA SANGUINIS HOMINIS.—Dr. Austin Flint, jr. (*New York Medical Journal*, June 15, 1895), reports a case of filariosis in a colored man, 22 years of age, from the West Indies, but residing for ten months in this country. His urine was milky, of a specific gravity of 1014, acid reaction, and contained albumen, but no sugar. Agitation with ether caused the urine to clear up, and under the microscope much fatty debris and fat globules, with some broken down red corpuscles, but no tube-casts, were found. The blood, examined shortly after midnight, showed the presence of filarial embryos. The patient complained, besides of this urinary condition, of headache, pain in the sacral region, and weakness, with some emaciation, and slight fever. Methylene blue (two grains every four hours) in a short while caused an abatement of all the symptoms, the urine becoming quite clear; but on discontinuing the remedy the symptoms all reappeared. The drug having been resumed, however, in five days more the case was permanently recovered. The use of the same drug in gonorrhœa and in malaria, is also noted in the paper, in connection with the discussion of the germicidal properties of the substance.

TREATMENT OF DIPHTHERIA WITH SOLUTION OF SUBSULPHATE OF IRON.—Dr. Wm. S. Stewart, of Philadelphia (*Medical and Surgical Reporter*, June 29, 1895), declares that in a practice of thirty-four years duration he has never lost a single case of uncomplicated diphtheria in an adult, and only one complicated one (the complication being in this case a severe croupous pneumonia); and he attributes his success to the employment of local and internal administration of Monsel's solution of ferric subsulphate. His experience with children has not been as gratifying, but nevertheless has been exceptionally successful. He would administer, at the earliest possible moment in the case, about seven grains of chlorate of potash and two drops of the subsulphate solution, which at times may cut short an attack. But when the membrane is well formed, these alone are valueless. Then a dilute solution (one fluid drachm of the Monsel solution to half a pint of cold or hot water, as the patient fancies) is used as a gargle, to be persisted in until every particle of the patch is gotten rid of. If the patient can not gargle, the parts must be swabbed with the solution until quite clean of membrane. The absolute removal of the membrane is the great necessity of the treatment. If this end be gained, Dr. Stewart does not fear the diphtheritic contagion in the least.

---

Dr. W. T. Sawyer, of Greensboro, Alabama, in a letter to the *Virginia Medical Monthly* of February, 1895, details several experiments and some practical experience in the use of the tincture of *asclepias verticellatta* (Linn.), var. *pumila* (Gray), in the treatment of poisoning from snake-bites, and too from the bites of rabid animals. The weed was suggested by the natives of the mountain lands of St. Clair county, Alabama, among whom a tincture has long been used in this class of cases, as well as for an intoxicating drink, its value having been originally made known by the Indians formerly inhabiting the district.

